# New opportunities and insights into *Papaver* self-incompatibility by imaging engineered Arabidopsis pollen

**DOI:** 10.1093/jxb/eraa092

**Published:** 2020-02-26

**Authors:** Ludi Wang, Marina Triviño, Zongcheng Lin, José Carli, Deborah J Eaves, Daniёl Van Damme, Moritz K Nowack, Vernonica E Franklin-Tong, Maurice Bosch

**Affiliations:** 1 Institute of Biological, Environmental and Rural Sciences (IBERS), Aberystwyth University, Plas Gogerddan, Aberystwyth, UK; 2 Department of Plant Biotechnology and Genetics, Ghent University, Ghent, Belgium; 3 VIB Center for Plant Systems Biology, Ghent, Belgium; 4 School of Biosciences, College of Life and Environmental Sciences, University of Birmingham, Edgbaston, Birmingham, UK; 5 University of Nottingham, UK

**Keywords:** Actin, actin-binding proteins (ABPs), calcium, endocytosis, fluorescent probes, programmed cell death (PCD), pH, pollen tube growth, live-cell imaging, self-incompatibility (SI)

## Abstract

Pollen tube growth is essential for plant reproduction. Their rapid extension using polarized tip growth provides an exciting system for studying this specialized type of growth. Self-incompatibility (SI) is a genetically controlled mechanism to prevent self-fertilization. Mechanistically, one of the best-studied SI systems is that of *Papaver rhoeas* (poppy). This utilizes two *S*-determinants: stigma-expressed PrsS and pollen-expressed PrpS. Interaction of cognate PrpS–PrsS triggers a signalling network, causing rapid growth arrest and programmed cell death (PCD) in incompatible pollen. We previously demonstrated that transgenic *Arabidopsis thaliana* pollen expressing PrpS–green fluorescent protein (GFP) can respond to *Papaver* PrsS with remarkably similar responses to those observed in incompatible *Papaver* pollen. Here we describe recent advances using these transgenic plants combined with genetically encoded fluorescent probes to monitor SI-induced cellular alterations, including cytosolic calcium, pH, the actin cytoskeleton, clathrin-mediated endocytosis (CME), and the vacuole. This approach has allowed us to study the SI response in depth, using multiparameter live-cell imaging approaches that were not possible in *Papaver*. This lays the foundations for new opportunities to elucidate key mechanisms involved in SI. Here we establish that CME is disrupted in self-incompatible pollen. Moreover, we reveal new detailed information about F-actin remodelling in pollen tubes after SI.

## Teaser

Use of genetically encoded fluorescent probes to monitor self-incompatibility (SI)-induced changes in pollen of the heterologous Arabidopsis ‘SI’ system has allowed multiparameter imaging and identified the involvement of clathrin-mediated endocytosis.

## Introduction

Pollination in higher plants is a crucial event, required for fertilization and seed set. The acceptance of compatible pollen and the subsequent steps leading to successful fertilization comprise a complex series of events involving interaction between the pollen and pistil (see Dresselhaus and Franklin-Tong, 2013). A compatible pollen grain landing on a stigma adheres and hydrates; if this is successful, it germinates and a pollen tube emerges. The pollen tube grows using polar tip growth to navigate its way to the ovules, interacting with the maternal reproductive tissues of the pistil, and transports the sperm cells to the ovules, where they effect fertilization. Over the last decade or so, our knowledge of molecular components involved in the interactions between pollen and pistil during both compatible and incompatible pollinations has increased extensively (see the recent review by Bedinger *et al.*, 2017).

Pollen tubes extend extremely rapidly (up to 1 cm h^−1^ in maize; Barnanas and Fridvalszky, 1984), providing a wonderful system to study this specialized type of cellular growth (Qin and Yang, 2011). Tip growth depends on polar exocytosis to deliver plasma membrane and cell wall material to the apical region (Hepler *et al.*, 2001; Yang, 2008; Luo *et al.*, 2017), and on clathrin-mediated endocytosis (CME), which internalizes excess membrane material deposited by exocytosis at the pollen tube tip (Grebnev *et al.*, 2017; Zhang *et al.*, 2019). Pollen grains can germinate to produce pollen tubes *in vitro*, providing a great experimental system to study the control of tip growth, without the complication of pistil tissues. Unlike most plant cells, which dedifferentiate and lose polarity when put into *in vitro* culture, pollen maintains its identity and polarity. While they do not grow to the lengths observed *in vivo*, *in vitro* grown pollen tubes grow relatively uniformly and exhibit highly polarized cytoplasmic organization with several zones, including an apical zone packed with vesicles, a cytoplasmic-rich nuclear zone, and a vacuolar zone further back (Hepler *et al.*, 2001). Cytoplasmic streaming moves organelles in a reverse fountain pattern and depends on an intact actin cytoskeleton (Staiger *et al.*, 2010). The accumulated knowledge so far reveals the existence of a complex, self-organizing signalling network, utilizing, amongst others, a tip-localized Rho GTPase, apical cytosolic Ca^2+^ gradients, reactive oxygen species (ROS), the actin cytoskeleton, and vesicular trafficking to supply cell wall and membrane components for pollen tube growth (see Qin and Yang, 2011 for a review).

In addition to mechanisms to maximize reproductive success, many higher plants utilize mechanisms to avoid self-fertilization. Self-incompatibility (SI) is the main genetic mechanism to prevent inbreeding. It is specified by *S*-determinant genes at a highly polymorphic, multiallelic *S*-locus; see Fujii *et al.* (2016). Here we describe the SI system in *Papaver rhoeas*, which has been well characterized, especially with respect to the downstream events triggered by the *S*-specific interaction (see reviews by Wilkins *et al*., 2014; Wang *et al.*, 2019). SI in *Papave*r utilizes two genetically linked *S*-determinants: a pollen-expressed transmembrane protein, PrpS, and a stigma-expressed secreted protein, PrsS. Interaction of cognate PrpS–PrsS results in rejection of incompatible pollen, triggering a signalling network causing rapid growth arrest and ensuing programmed cell death (PCD) in incompatible pollen. This SI system serves as a good model system to investigate various ways in which pollen tube tip growth can be prevented, thereby providing insights into mechanisms involved in regulating pollen tube growth. Below we describe in more detail key features of the *Papaver* SI system that are relevant to the current study.

A key feature of *Papaver* SI is the inhibition of pollen tube growth. One of the first events observed after a cognate PrpS–PrsS interaction is a loss of the apically focused gradient of cytosolic free Ca^2+^ ([Ca^2+^]_cyt_), typical of growing pollen tubes (Hepler *et al.*, 2001), and a concomitant rapid increase in [Ca^2+^]_cyt_ in the shank of the pollen tube (Franklin-Tong *et al.*, 1993, 1995, 1997). More recently, evidence for Ca^2+^ influx has been obtained (Wu *et al.*, 2011). Downstream, SI triggers rapid increases in ROS in the pollen tube shank (Wilkins *et al.*, 2011, 2014). SI also triggers dramatic acidification of the cytosol ([pH]_cyt_), with the pH dropping rapidly to 6.5 within 10 min, reaching pH 5.5 by ~60 min (Wilkins *et al.*, 2015).

However, *Papaver* SI does not end with just inhibition of pollen tube growth. The SI-induced signalling cascade also triggers PCD involving a DEVDase/caspase-3-like activity that is activated several hours after the initial cognate interaction (Thomas and Franklin-Tong, 2004; Bosch and Franklin-Tong, 2007). This enzyme has a pH optimum of ~5.5 and is inactive at normal physiological pH of ~6.8 (Bosch and Franklin-Tong, 2007; Wilkins *et al.*, 2015). The cytosolic acidification induced by SI fits well with the optimal activity of this caspase-like enzyme. Indeed, data have shown that cytosolic acidification is necessary and sufficient to trigger PCD in *Papaver* pollen tubes (Wilkins *et al.*, 2015), suggesting that this is an important decision-making step in SI-PCD.

The actin cytoskeleton, which plays a key role in many cellular events, including tip growth, morphogenesis, and vesicle trafficking, is a key target of SI in *Papaver*. A cognate PrpS–PrsS interaction triggers rapid actin depolymerization, sufficient to inhibit pollen tube growth, which relies on an intact F-actin network (Snowman *et al.*, 2002). Downstream of this SI-induced actin depolymerization, further major alterations in actin localization and dynamics are observed, involving the formation of ‘punctate foci’; these are unusually stable actin structures and are a key feature of SI pollen (Poulter *et al.*, 2010). SI-induced acidification plays a key role in mediating actin remodelling. Artificial manipulation of the [pH]_cyt_ of pollen tubes revealed that pH 5.5 triggered the formation of numerous F-actin foci, while buffering to pH 7, to prevent SI-induced acidification, blocked the formation of these actin foci (Wilkins *et al.*, 2015). This demonstrated that acidification was required for these actin alterations. Studies in other eukaryotic cells have shown that changes in cytoskeletal dynamics can influence whether PCD is initiated (see reviews by Franklin-Tong and Gourlay, 2008; Smertenko and Franklin-Tong, 2011). In *Papaver*, experiments using actin depolymerization and stabilization treatments implicated that both types of actin alterations can trigger a caspase-3 like/DEVDase activity. This, together with other data, provided evidence that actin alterations are implicated in not only inhibition of pollen tube growth, but also in mediating PCD (Thomas *et al.*, 2006; Bosch and Franklin-Tong, 2008).

Actin-binding proteins (ABPs) regulate virtually every aspect of the actin cytoskeleton, including its assembly, turnover, dynamics, and organization (Staiger *et al.*, 2010; Pollard, 2016). In incompatible *Papaver* pollen, the SI-induced actin foci are associated with at least two ABPs: actin-depolymerizing factor (ADF/cofilin) and cyclase-associated protein (CAP/Srv2p; Poulter *et al.*, 2010), suggesting their involvement in the formation of these highly stable F-actin foci observed during SI in incompatible pollen.

Recent studies have demonstrated the functional transfer of PrpS and PrsS to *Arabidopsis thaliana*, rendering these plants fully self-incompatible, with little or no seed set (Lin *et al.*, 2015), despite Arabidopsis being highly diverged (>140 million years) from *Papaver*. A previous study showed that addition of recombinant PrsS to Arabidopsis pollen expressing PrpS resulted in key features of the *Papaver* pollen SI response, including formation of actin foci and increases in DEVDase/caspase-3-like activity (de Graaf *et al.*, 2012). These data suggested that Arabidopsis pollen has the cellular machinery required for the activation of downstream events triggered by PrpS–PrsS interaction. The successful functional transfer of the *Papaver* SI system to Arabidopsis (de Graaf *et al.*, 2012; Lin *et al.*, 2015) provides exciting opportunities to further dissect the SI-induced signalling networks and events leading to PCD in this model system. Here we describe recent advances in utilizing the engineered Arabidopsis ‘SI’ system in combination with a range of genetically encoded probes that allows us to study the mechanistic basis of the *Papaver* SI-PCD system in depth. This provides new opportunities and directions to further elucidate and dissect key mechanisms and components involved in SI-PCD.

## Materials and methods

### Plant material and growth conditions

*Arabidopsis thaliana* accession Columbia-0 (Col-0) seeds and those from derived transgenic lines were grown at 22 °C in a 16 h light/8 h dark cycle. Pollen grains from mature flowers of the marker lines were used (see Supplementary Table S1 and Supplementary Fig. S1 at *JXB* online).

### Growth of Arabidopsis pollen tubes and treatments

Arabidopsis pollen was hydrated for 50 min in 35 mm glass-bottom microwell culture dishes with a 10 mm No. 1.5 coverglass (MatTek Corp.) coated with 0.01% (w/v) poly-l-lysine. Hydrated pollen was grown in liquid germination medium (GM) comprising 15% (w/v) sucrose, 0.01% (w/v) H_3_BO_3_, 5 mM KCl, 1 mM MgSO_4_, 2.5 mM CaCl_2_, and 2.5 mM Ca(NO_3_)_2_ (modified from de Graaf *et al.*, 2012) for >60 min prior to treatment. Recombinant PrsS_1_ proteins were produced as described previously (Wilkins *et al.*, 2015). SI was induced by adding recombinant PrsS_1_ with a final concentration of 20 μg ml^−1^. Controls were treated with GM or heat-inactivated PrsS_1_. Manipulation of cytosolic pH was achieved as described by Wilkins *et al.* (2015). PrsS_1_ and the Ac-DEVD-AMC probe (1.5 mM final concentration) were simultaneously added. For treatments that included Ac-DEVD-CHO (100 µM final concentration), this was added at the same time as PrsS_1_ and Ac-DEVD-AMC.

### *In vitro* test of PrsS_1_ activity

Pollen tubes were grown and treated as described above. Pollen tubes were imaged immediately after, and 2 h after SI treatment, using a Leica DMi8 microscope equipped with a Leica TCS SPE camera. Pollen tube lengths [20 pollen tubes per treatment for each experiment, three independent experiments (*n*=60 for each treatment)] were measured using Fiji software (Schindelin *et al.*, 2012).

### Reverse transcription–PCR analysis of *PrpS*_*1*_ expression level in Arabidopsis lines

*PrpS*_*1*_ expression in Arabidopsis pollen from transgenic PrpS_1_–green fluorescent protein (GFP), YC3.6_PrpS_1_, pHGFP_PrpS_1_, and PrpS_1_ lines was analysed using reverse transcription–PCR (RT–PCR) on cDNA prepared from mature flowers. Total RNA was extracted using TRIzol reagent (Life Technologies) and purified using RNeasy MiniElute Cleanup Kit columns (Qiagen). After DNase I treatment (New England Biolabs), isolated total RNA was used for cDNA synthesis (SuperScript^®^ III First-Strand Synthesis System, Life Technologies) followed by PCR using *PrpS*_*1*_ gene-specific primers (see Supplementary Table S2). *Glyceraldehyde-3-phosphate dehydrogenase* (*GAPD*) primers were used as a positive control.

### FM4-64 internalization assays

FM4-64 was added to pollen tubes expressing PrpS_1_–GFP at different time points after SI induction to a final concentration of 3 μM and incubated for 20 min prior to imaging. The FM4-64 signal was observed with a Leica SP8 confocal microscope equipped with a ×100 CS2 oil objective (NA 1.40, excitation 561 nm, emission 600–680 nm). FM4-64 signal intensity was measured using Fiji. Regions of interest (ROIs) were selected based on the plasma membrane or cytosol area 15–25 μm from the apex.

### Visualization of TPLATE–TagRFP, TPLATE–GFP, and CLC2–TagRFP

Pollen tubes from plants harbouring *pLAT52::TPLATE-TagRFP_pNTP303::PrpS*_*1*_*-GFP* (homozygous *tplate*/*tplate* background) and *pLAT52::TPLATE-GFP* (*tplate*/*tplate*)*_pRPS5A::CLC2(At2g40060)-TagRFP* (Supplementary Table S1A; Gadeyne *et al.*, 2014; see Supplementary Protocols) were observed using a Leica SP8 confocal microscope (×100 CS2 objective, NA 1.40); red fluorescent protein (RFP) excitation 561 nm, emission 571–700 nm; GFP excitation 488 nm. Fluorescence intensity was obtained using the standard profiling function in Fiji.

### Ratio-imaging of cytosolic Ca^2+^

Pollen tube [Ca^2+^]_cyt_ was imaged using a Leica SP8 confocal microscope (×100 CS2 objective, NA 1.40). The YC3.6 Ca^2+^ sensor (Nagai *et al.*, 2004) was excited at 458 nm; emissions of cyan fluorescent protein (CFP; 465–500 nm) and Venus (520–570 nm) were collected simultaneously. Images were processed using Leica Application Suite X (LAS X).

### Visualization and image analyses of F-actin

Lifeact-mRuby2 (Riedl *et al.*, 2008; Bascom *et al.*, 2018) was used to visualize the F-actin cytoskeleton. For the ‘slow’ SI line, Lifeact-mRuby2 was introduced by transformation of *pNTP303::Lifeact-mRuby2* into Arabidopsis plants harbouring *pNTP303::PrpS*_*1*_*-GFP* as described previously (de Graaf *et al.*, 2012). For the ‘fast’ lines, Lifeact-mRuby2 was introduced by crossing Arabidopsis plants expressing Lifeact-mRuby2 with lines harbouring *pNTP303::YC3.6-pNTP303::PrpS*_*1*_ or *pNTP303::pHGFP-pNTP303::PrpS*_*1*_ (Table S1). F-actin labelled with Lifeact-mRuby2 was observed using a Leica SP8 confocal microscope (×100 CS2 objective, NA 1.40, excitation 561 nm). A kymograph with a line thickness of 1 pixel along the area adjacent to the plasma membrane of pollen tubes was generated to analyse the intensity of F-actin at the pollen tube cortex and pseudocolour-processed using Fiji (Schindelin *et al.*, 2012). The distribution of F-actin filament orientation was analysed by using the ImageJ/Fiji plugin ‘OrientationJ’ (Rezakhaniha *et al.*, 2012; Püspöki *et al.*, 2016).

### Ratio-imaging of pHGFP

The cytosolic pH sensor pHGFP was sequentially excited at 405 nm and 488 nm. For each of the excitations, the emission was collected separately between 500 nm and 530 nm. Using LAS X software, the fluorescent intensity ratios from the 405 nm and 488 nm excitation channels (R_405/488_) were quantified in an ROI of 5×20 μm covering an area 15–35 μm distal from the apex. A standard curve defining pH values relative to the R_405/488_ was generated after each imaging session by measuring R_405/488_ in pollen tubes treated with 50 mM propionic acid pH 7.5, 7.0, 6.5, 6.0, and 5.5, respectively.

### Analysis of vacuolar morphology

Pollen grains expressing PrpS_1_–GFP and the tonoplast fluorescent marker WAVE9R, containing mCherry attached to VAMP711 (Geldner *et al.*, 2009; Supplementary Table S1), were analysed for vacuolar morphology. Arabidopsis lines carrying both *pNTP303::PrpS*_*1*_*-GFP* and *pUBQ10::VAMP711-mCherry* were obtained by crossing and selection of lines positive for both cassettes. Mid-plane images of pollen tubes were acquired using a Leica SP8 confocal microscope (×63 CS2 objective, NA 1.20, excitation 561 nm, emission 576–680 nm).

### Analysis of PCD

The fluorescent probe Ac-DEVD-AMC was used for the detection of SI-induced DEVDase activity in pollen grains expressing PrpS_1_. Pollen samples were incubated in the dark and images were taken every 30 min using a Leica SP8 confocal microscope (×63 CS2 objective, NA 1.20, excitation 405 nm, emission 451–496 nm). For each time point, 100–300 pollen grains were scored in three independent experiments. The AMC signal was quantified using LAS X software, based on the mean fluorescence intensity values from a 15 µm diameter ROI, drawn over individual pollen grains. *P*-values were calculated using the Wilcoxon test. Nuclear disruption was assessed using Arabidopsis pollen expressing PrpS_1_–GFP and NLS (nuclear localization signal)–tdTomato (Supplementary Table S1); ×63 CS2 objective, NA 1.20, excitation 561 nm, emmission 596–665 nm.

## Results

### PrsS–PrpS interaction triggers inhibition of Arabidopsis pollen tube growth

**Fig. 1.**
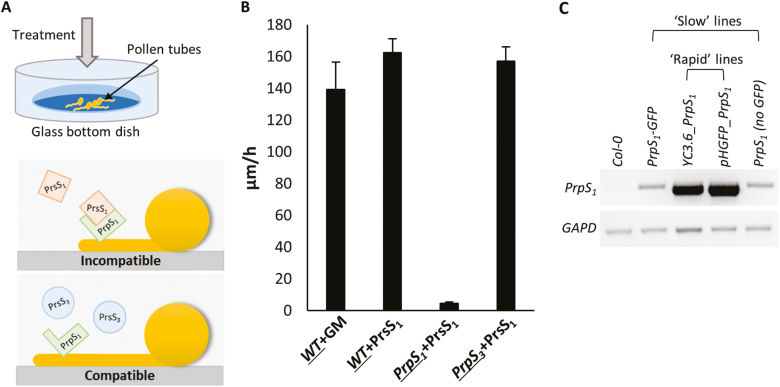
*S*-specific inhibition of *in vitro* growth of transgenic Arabidopsis pollen tubes undergoing an incompatible response. (A) Cartoon illustrating the *in vitro* pollen tube growth system in liquid germination medium (GM). Treatment of a transgenic Arabidopsis pollen tube expressing *PrpS*_*1*_ with recombinant PrsS_1_ triggers inhibition, as it is an incompatible combination, but when PrsS_3_ is added to a pollen tube expressing *PrpS*_*1*_ (a compatible combination), pollen tube growth is not inhibited. (B) *In vitro* growth rates of pollen tubes. Wild-type (WT) pollen tubes treated with GM or recombinant PrsS_1_ have normal growth rates; pollen tubes expressing *PrpS*_*3*_ treated with recombinant PrsS_1_ are compatible and display normal growth rates; pollen tubes expressing *PrpS*_*1*_ treated with recombinant PrsS_1_ are incompatible and pollen tube growth is arrested (*n*=60). The pollen genotype is underlined. (C) Semi-quantitative RT–PCR shows the different expression levels of *PrpS*_*1*_ in transgenic *A. thaliana* lines harbouring constructs containing *PrpS*_*1*_ used in this study. *GAPD* was used as an internal control. ‘Slow’ and ‘rapid’ refer to the speed of the SI response associated with low and high *PrpS*_*1*_ expression levels, respectively.

Pollen tubes from the engineered Arabidopsis ‘SI’ system plants were grown *in vitro* and either recombinant PrsS proteins were added to stimulate SI or GM was added as a control (see the Materials and methods; Fig. 1A). These *in vitro* assays confirmed the *S*-specific activity of PrsS_1_, as pollen tube growth rates were significantly inhibited when recombinant PrsS_1_ was added to pollen tubes expressing PrpS_1_–GFP (a cognate combination triggering SI, Fig. 1B; Supplementary Movie S1). No growth inhibition was observed for wild-type pollen tubes treated with GM or PrsS_1_. Likewise, pollen tubes expressing PrpS_3_–GFP treated with PrsS_1_ (a compatible combination) were not inhibited. These data correspond to the previously reported SI response in PrpS-expressing Arabidopsis pollen (de Graaf *et al.*, 2012), and serve as a baseline to confirm the biological activity of PrpS_1_ and the *S*-specific incompatible response triggered. Arabidopsis lines expressing PrpS together with various fluorescent genetic markers varied in their responses. Semi-quantitative RT–PCR analysis of the lines (Supplementary Table S1) linked this variation with differences in *PrpS*_*1*_ expression levels in these lines (Fig. 1C); we will describe the effect of this later.

### PrpS–PrsS interaction triggers alterations in clathrin-mediated endocytosis

**Fig. 2.**
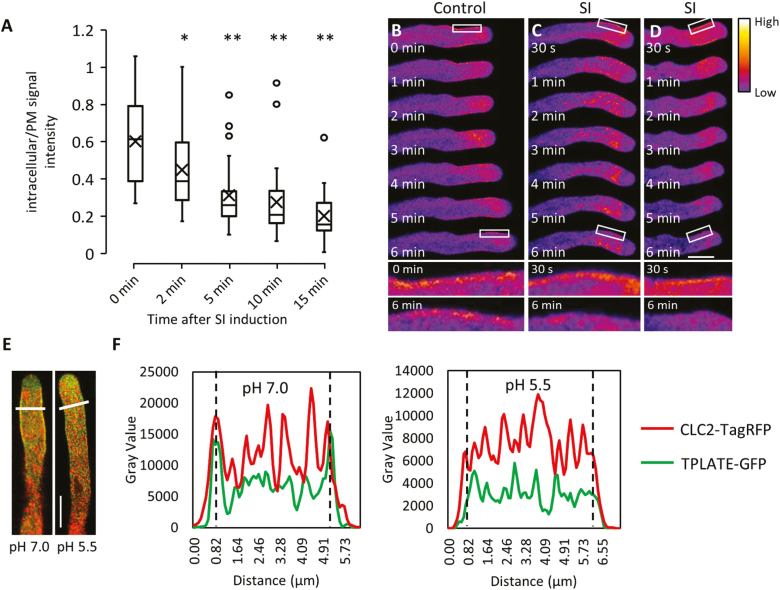
CME is inhibited after SI induction and cytosolic acidification. (A) Boxplots showing quantification of the FM4-64 signal intensity as a ratio (in the cytoplasm versus on the plasma membrane) at different time points after SI induction. Significance levels are based on comparisons with ‘0 min’. *P<0.01 and **P<0.001 with Kruskal–Wallis ANOVA on ranks (*n*=34, 31, 30, 28, and 27 for time points 0, 2, 5, 10, and 15 min, respectively). (B) Time-lapse fluorescent images showing the localization of TPLATE–TagRFP at the subapical plasma membrane region of a growing pollen tube; see also higher magnification images of selected areas (white box) after 0 min and 6 min. (C and D) After SI induction, the localization of TPLATE–TagRFP at the plasma membrane is rapidly lost; see also higher magnification images after 6 min. The position of the RFP signal on the plasma membrane immediately after treatment is clearly visible at higher magnification. The fluorescence intensity of TPLATE–TagRFP is indicated by the colour gradient. Corresponding fluorescence intensity profiles are shown in Supplementary Fig. S2. Scale bar=10 μm. (E) Representative images showing the co-localization of TPLATE–GFP (green) and CLC2–TagRFP (red) at the plasma membrane at cytosolic pH 7.0 while plasma membrane recruitment is abolished at pH 5.5. Scale bar=10 μm. (F) Fluorescence intensity profiles for the pollen tubes along the white lines indicated in (E). Dashed lines indicate the position of the plasma membrane.

CME is important in mediating pollen tube growth (Grebnev *et al.*, 2017). However, whether SI affects pollen tube membrane internalization has not previously been established. To monitor the dynamics of CME during the SI response, we visualized CME by using the internalization of the endocytic tracer dye FM4-64 as a readout of altered endocytosis, and TPLATE, as a member of the endocytic TPLATE complex (TPC), as an indicator for CME (Gadeyne *et al.*, 2014). The uptake of FM4-64 into pollen tubes was significantly inhibited within 5 min after SI induction (Fig. 2A), which provides evidence that endocytosis is inhibited very early during the SI response. This block in internalization thus occurs concomitantly with SI-induced inhibition of pollen tube growth.

TPLATE is one of the eight subunits of TPC, which functions as a crucial plasma membrane-localized adaptor complex for CME in plants (Van Damme *et al.*, 2011; Gadeyne *et al.*, 2014). Similar to what has been shown for TPLATE–GFP (Van Damme *et al.*, 2006), TPLATE–TagRFP is recruited to the plasma membrane in the subapical area of growing pollen tubes (Fig. 2B; Supplementary Fig. S2). This localization of TPLATE is in agreement with observations of another TPC subunit, TML, identifying the subapical plasma membrane region as the site of CME during pollen tube growth (Gadeyne *et al.*, 2014). Within 2 min after SI induction, TPLATE no longer localized at the plasma membrane (Fig. 2C, D). These results provide evidence that CME is rapidly inhibited early during the SI response.

As CME dynamics in Arabidopsis depend on cytoplasmic pH (Dejonghe *et al.*, 2016), this suggested that SI-induced cytosolic acidification (Wilkins *et al.*, 2015; Fig. 5) may affect CME in pollen tubes. To investigate the effect of low cytosolic pH on CME in pollen tubes, we used propionic acid to manipulate pollen tube [pH]_cyt_ (Wilkins *et al.*, 2015). TPLATE–GFP and CLC2–TagRFP (Gadeyne *et al.*, 2014) maintained their localization at the plasma membrane of the subapical area of pollen tubes with propionic acid pH 7.0 (Fig. 2E, F). The recruitment of TPLATE–GFP and CLC2–mTagRFP at the plasma membrane was disrupted by the addition of propionic acid pH 5.5 (Fig. 2E, F). As a functional TPC, together with CLC2 at the plasma membrane, is required for CME (Van Damme *et al.*, 2011; Gadeyne *et al.*, 2014), our observations suggest that CME is altered and inhibited by acidic cytosolic conditions.

### PrsS–PrpS interaction triggers increases in cytosolic Ca^2+^

**Fig. 3.**
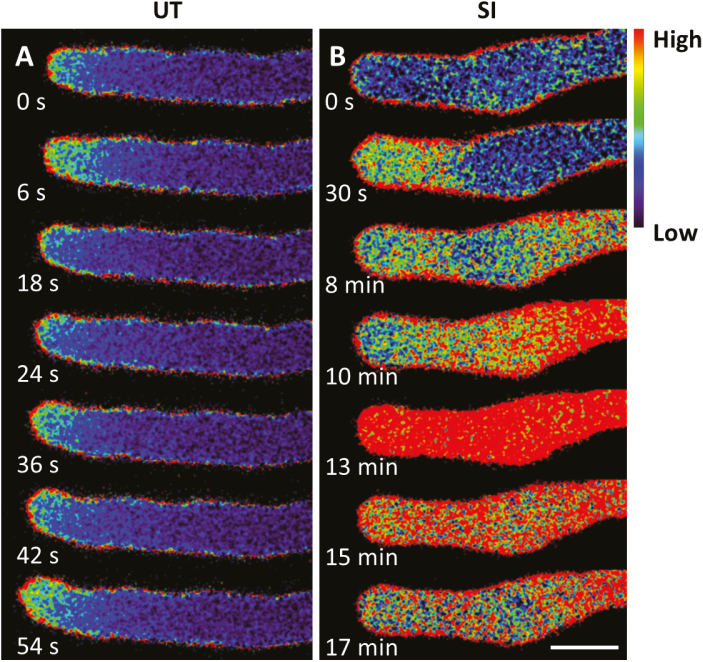
Cytosolic free Ca^2+^ increases in Arabidopsis pollen tubes during the SI response. Representative ratio-imaging examples of Arabidopsis pollen tubes expressing *PrpS*_*1*_ and *YC3.6* without treatment (A, untreated, UT) and after treatment with PrsS_1_ (B, SI). Scale bar=10 μm.

To determine whether PrpS-expressing Arabidopsis pollen tubes undergo similar [Ca^2+^]_cyt_ alterations to *Papaver* pollen tubes during the SI response, we used the genetically encoded fluorescent Ca^2+^ sensor YC3.6 (Nagai *et al.*, 2004) to monitor alterations in [Ca^2+^]_cyt_. Ratio-imaging of untreated growing pollen tubes co-expressing PrpS_1_ and YC3.6 revealed a tip-focused [Ca^2+^]_cyt_ gradient associated with tip growth (Fig. 3A), previously observed in many pollen tubes growing *in vitro*, including *Papaver* and Arabidopsis pollen tubes (Franklin-Tong *et al.*, 1997; Hepler *et al.*, 2001; Iwano *et al.*, 2009). After addition of PrsS_1_ to induce the SI response, ratio-imaging revealed that the apical [Ca^2+^]_cyt_ gradient rapidly dissipated (within 1 min), and a more dispersed area of elevated [Ca^2+^]_cyt_ was observed in the general tip region (Fig. 3B). This area of elevated [Ca^2+^]_cyt_ extended further back into the shank area at 8 min. Ten minutes after SI induction, a dramatic increase of [Ca^2+^]_cyt_ was detected in the shank area and appeared to peak at 13 min (Fig. 3B). Subsequently, the [Ca^2+^]_cyt_ decreased. These results are similar to the SI-induced spatio-temporal changes for [Ca^2+^]_cyt_ reported in *Papaver* pollen tubes (Franklin-Tong *et al.*, 1997). This demonstrates that the genetically encoded marker YC3.6 allows us to monitor and describe the [Ca^2+^]_cyt_ signature during the SI response.

### PrsS–PrpS interaction triggers actin remodelling

**Fig. 4.**
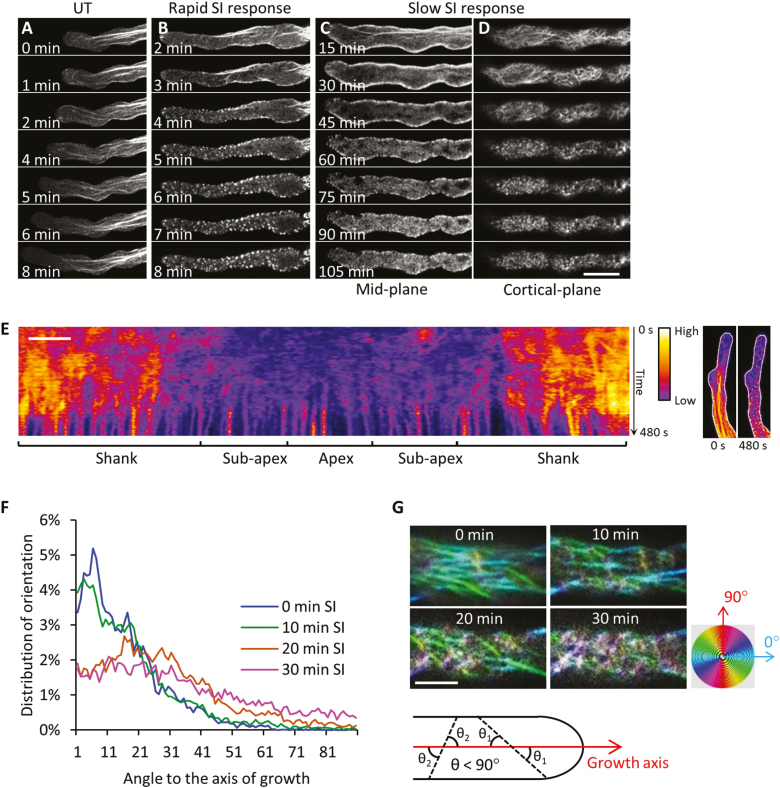
F-actin remodelling in Arabidopsis pollen tubes during the SI response. (A–D) Time-lapse images showing SI-induced remodelling of the F-actin cytoskeleton using Lifeact-mRuby2 as a marker, in pollen tubes of two Arabidopsis lines with different expression levels of *PrpS*_*1*_. (A) The F-actin cytoskeleton of an untreated growing pollen tube. (B) Remodelling of the F-actin cytoskeleton during an SI response in an Arabidopsis ‘rapid’ line with a high level of expression of *PrpS*_*1*_. (C) Mid-plane images of a time-lapse sequence showing remodelling of the F-actin cytoskeleton during an SI response in an Arabidopsis ‘slow’ line with a low level of expression of *PrpS*_*1*_. (D) Cortical plane images of the pollen tube shown in (C). (E) Pseudocoloured kymograph analysis of F-actin (monitored with Lifeact-mRuby2) adjacent to the plasma membrane of a representative pollen tube (maximum projection) during the SI response in a ‘rapid’ line. The fluorescence intensity in the kymograph indicates the amount of F-actin near the plasma membrane of the pollen tube (shown by white lines in the images on the right). The fluorescence intensity is indicated by the colour gradient. Scale bar=5 μm. (F) Alterations in the distribution of the angles between F-actin filaments and the growth axis of the pollen tube at different time points after SI induction. (G) Representative images show the rearrangement of F-actin filament arrays at the cortical region of pollen tubes during the early stages of SI. The spectrum disc indicates the pseudocolours applied to actin filaments based on their orientations relative to the growth axis of the pollen tube. The diagram demonstrates the angles (θ _1_, θ _2_ …) between actin filaments (dashed lines) and the growth axis (red arrow) of the pollen tube. Only the sharp angles (θ<90°) were measured. Scale bar=10 μm.

Dramatic SI-induced remodelling of the F-actin cytoskeleton has been described in both incompatible *Papaver* pollen tubes (Geitmann *et al.*, 2000; Snowman *et al.*, 2002; Poulter *et al.*, 2010) and transgenic Arabidopsis pollen tubes expressing PrpS (de Graaf *et al.*, 2012). However, previous data used fixed pollen tubes from a few time points and relied on the staining of F-actin with phalloidin. Here we used the genetically encoded filamentous actin probe Lifeact-mRuby2 (Riedl *et al.*, 2008; Bascom *et al.*, 2018) to initiate investigations into the dynamic remodelling of the actin cytoskeleton in incompatible pollen tubes using live-cell imaging. In an untreated pollen tube, the F-actin arrays comprise filament bundles that are arranged longitudinally and parallel to the growth axis, and pollen tube growth is evident (Fig. 4A). After SI induction, the F-actin cytoskeleton rapidly altered and formation of punctate F-actin foci, a signature event of the SI response, was observed (Fig. 4B–D; Supplementary Movie S2).

Different Arabidopsis lines expressing PrpS_1_ with Lifeact-mRuby2 together with other markers varied in their response times. Pollen tubes with high expression levels of PrpS_1_ exhibited a more rapid response compared with lines with much lower expression levels of PrpS_1_ (Fig. 1C; Supplementary Table S1); we named these ‘rapid’ and ‘slow’ lines, respectively. In a ‘rapid’ transgenic Arabidopsis line, incompatible pollen tubes co-expressing PrpS_1_ and Lifeact-mRuby2 exhibited a rapid SI response, and F-actin foci were usually observed within 10 min after SI induction (Fig. 4B; Supplementary Movie S2). Within a few minutes of SI induction in a representative ‘rapid’ pollen tube, the longitudinal F-actin bundles disappeared, while predominant cortical F-actin bundles adjacent to the plasma membrane remained. From ~4–5 min onwards, F-actin foci started to form (Fig. 4B). This rapidly reacting line enabled us to observe the entire process of actin remodelling during SI, allowing observation of the progression of actin filaments into foci structures (Supplementary Movie S2), which could not be achieved with conventional fixation approaches.

To further dissect the actin dynamics in incompatible pollen tubes, a ‘slow’ transgenic Arabidopsis line was utilized to obtain spatio-temporal details of the F-actin reorganization (Fig. 4C–D, F–G). Mid-plane images of a representative pollen tube (Fig. 4C) revealed that even 15 min after SI induction, small numbers of thick actin filament bundles were still apparent; these disappeared ~30 min after SI induction. Actin filaments in the cortical region appeared to become shorter and dramatically reorganized, forming a ‘fishing net’ pattern, suggesting that they were severed (Fig. 4D). By 60 min, the actin population in this cortical region fragmented further, appearing to form small foci. Later, these F-actin foci increased in size.

To characterize the F-actin remodelling during SI further, we used kymograph analysis to monitor the SI-induced changes in dynamics of F-actin at the cortex of pollen tubes from a ‘rapid’ line (Fig. 4E; Supplementary Movie S2). This shows two distinct areas: one area apparent during early SI, with a rather random signal that shows highly dynamic F-actin adjacent to the plasma membrane; and another area later (after 6–7 min), appearing as multiple straight lines. This latter observation shows that the punctate actin foci are not dynamic and do not move (Fig. 4E). As the transition between these two areas is quite distinct, it shows that the change from dynamic F-actin filament arrays to highly stable F-actin foci is rapid. This reveals that (at least at the cortical region of these rapidly responding pollen tubes), the formation of foci is a sudden event rather than a gradual development. Further analyses will be needed to determine if formation of foci in the centre of pollen tubes is similar to that in the cortical region.

To characterize the distinctive patterns formed by F-actin in the cortical region during the early stages of the SI response, we quantified the orientation of actin filaments at different time points during the first 30 min after SI induction in a ‘slow’ line (Fig. 4F). At time 0, the orientation of F-actin bundles was mostly parallel to the growth axis, predominantly distributed between 0° and 30° relative to the growth axis (Fig. 4F). Imaging at 10 min after SI induction showed that a small proportion of actin bundles had reorientated relative to the growth axis (Fig. 4G). After 20 min, the F-actin bundles reoriented further to form the ‘fishing net’ pattern (Fig. 4G), reflected by the wider distribution of angles (Fig. 4F). After 30 min, F-actin bundles appeared to be randomly orientated relative to the growth axis (Fig. 4G) with angles distributed throughout 0–90° (Fig. 4F). These observations begin to describe the actin remodelling during the SI response.

### PrpS–PrsS interaction triggers decreases in cytosolic pH

**Fig. 5.**
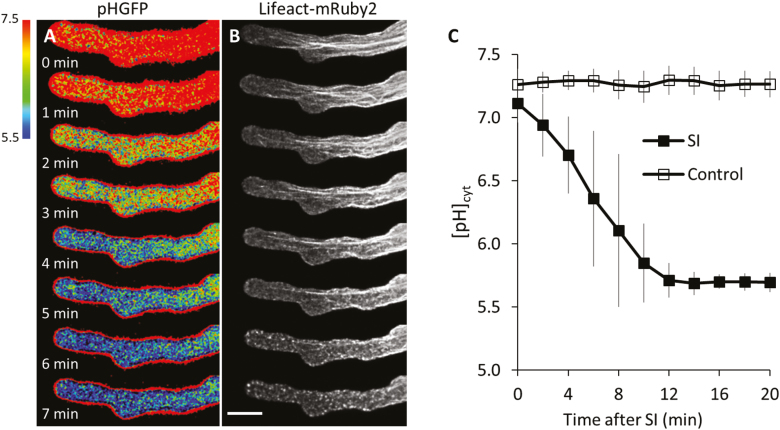
Rapid cytosolic acidification and remodelling of the actin cytoskeleton in Arabidopsis pollen tubes during the SI response. (A) Representative time-lapse ratio-images of pHGFP in pollen tubes of a ‘rapid’ line after treatment with PrsS_1_ to induce SI. (B) Remodelling of F-actin visualized with Lifeact-mRuby2 in the same pollen tube as shown in (A) during the SI response. Scale bar=10 μm. (C) Quantification of the changes in cytosolic pH after SI induction (SI; *n*=20) or treatment with inactivated PrsS_1_ (control; *n*=10). Error bars indicate the SD.

The PrpS–PrsS interaction triggers dramatic intracellular acidification which acts as an important regulatory factor of the SI response in *Papaver* (Bosch and Franklin-Tong, 2007; Wilkins *et al.*, 2015; Wang *et al.*, 2019). To further investigate the regulatory role of cytosolic acidification in *Papaver* SI-PCD, we made a transgenic Arabidopsis ‘SI’ line co-expressing Lifeact-mRuby2 and a pH indicator, pHGFP (Moseyko and Feldman, 2001). This ‘rapid’ line (Fig. 4E) enabled us to capture SI-induced changes in [pH]_cyt_ and remodelling of F-actin in the same pollen tube (Fig. 5A, B). Two minutes after SI induction, the [pH]_cyt_ rapidly decreased, while thick F-actin bundles in the central region of the pollen tube remained. Four minutes after SI induction, the [pH]_cyt_ further dropped to a mean value of approximately pH 6.0 (Fig. 5A), and small F-actin foci started to be visible in the region close to the tip (Fig. 5B). By 7 min, the mean [pH]_cyt_ had decreased further (Fig. 5A) and large F-actin foci had formed and were distributed throughout the pollen tube (Fig. 5B). The extent of SI-induced cytosolic acidification, determined using this genetically encoded pH indicator (Fig. 5C), was similar to that previously reported for incompatible *Papaver* pollen tubes (Wilkins *et al.*, 2015), although the [pH]_cyt_ decreased faster (Fig. 1C), probably due to the high expression level of PrpS_1_. The ability to monitor [pH]_cyt_ and F-actin within the same pollen tube provides a valuable tool to investigate mechanistic links between cytosolic pH and dynamic remodelling of the actin cytoskeleton during the SI response in the future.

### PrpS–PrsS interaction triggers co-localization of CAP1 and ADF7 with F-actin foci

**Fig. 6.**
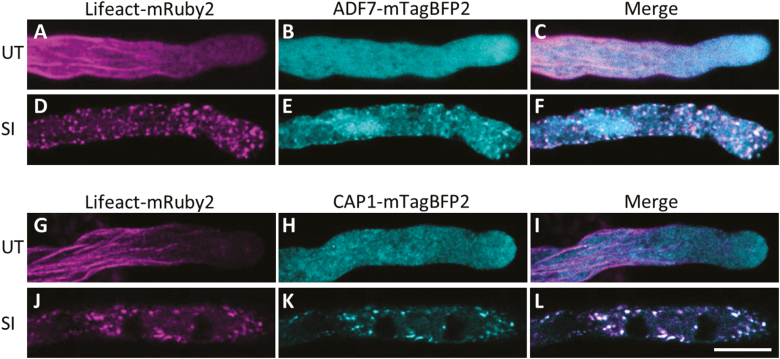
SI induction leads to the co-localization of ADF7 and CAP1 with F-actin foci. ADF7–mTagBFP2, CAP1–mTagBFP2 (both in cyan), and F-actin localization (Lifeact-mRuby2 in magenta) are shown in untreated pollen tubes expressing *PrpS*_*1*_ (A–C and G–I) or after SI induction (D–F and J–L). In untreated pollen tubes, ADF7 and CAP1 were distributed throughout the cytoplasm, with no major co-localization with F-actin (C and I). After SI induction, ADF7 and CAP1 co-localized with F-actin foci (F and L, respectively). Co-localization shows as white. Scale bar=10 μm.

In *Papaver*, two ABPs, CAP and ADF, co-localized with F-actin foci after SI induction and after artificially lowering the [pH]_cyt_ to 5.5 (Poulter *et al.*, 2010, Wilkins *et al.*, 2015). This suggested that they may play important roles in the formation of foci. We generated ‘SI’ Arabidopsis lines co-expressing fluorescently tagged Arabidopsis CAP1 and ADF7: CAP1–mTagBFP2 or ADF7–mTagBFP2 with Lifeact-mRuby2, which enabled us to simultaneously detect the respective ABP and F-actin (Fig. 6). In untreated pollen tubes, ADF7–mTagBFP2 was distributed homogenously throughout the pollen tube cytosol (Fig. 6B). CAP1–mTagBFP2 showed a similar distribution, however with a slightly more speckled appearance. Two hours after SI induction, both ABPs had formed structures that resemble F-actin foci (Fig. 6D, E, J, K), and both ABPs co-localized with the F-actin foci (Fig. 6F, L). These live-cell observations correspond to previous findings in fixed and stained *Papaver* pollen tubes (Poulter *et al.*, 2010), providing good evidence that transgenic Arabidopsis ‘SI’ pollen tubes undergo a similar SI response. This suggests that these ABPs are likely to play mechanistic roles in mediating actin dynamics and foci formation during SI. Thus, this new approach of dual labelling of ABPs and F-actin with live-cell imaging provides us with powerful tools to help establish the functional roles of CAP1 and ADF7 in SI-PCD in the future.

### PrpS–PrsS interaction triggers alterations in vacuolar morphology

**Fig. 7.**
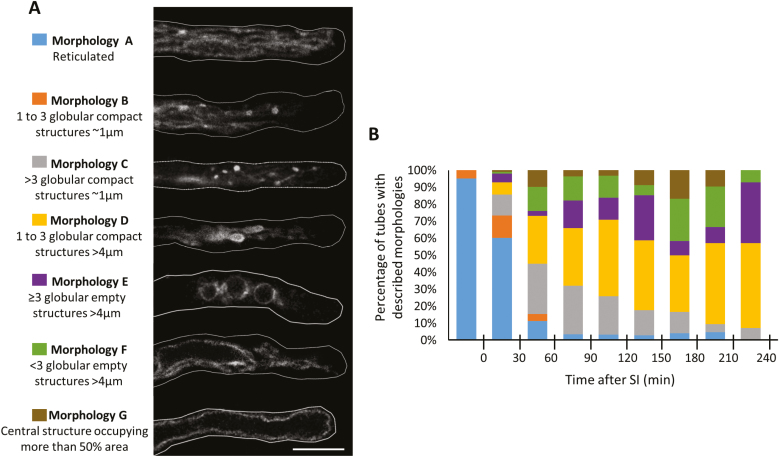
Changes in vacuolar morphologies in pollen tubes during the SI response. (A) Categorization and descriptions of different vacuolar morphologies observed in transgenic Arabidopsis pollen tubes after SI induction. The different morphologies were categorized according to the distribution of VAMP711–mCherry, a vacuolar membrane-localized protein, in the median focal plane of the pollen tubes. Scale bar=10 µm. (B) Quantification of pollen tubes exhibiting the vacuolar morphologies shown in (A) at different time points during the SI response.

Previous studies revealed reorganization of the vacuole in *Papaver* pollen tubes after SI induction (Wilkins *et al.*, 2015; Wang *et al.*, 2019). Here we used Arabidopsis ‘SI’ pollen expressing VAMP711–mCherry (WAVE9R; Geldner *et al.*, 2009), which localizes to the vacuolar membrane, to monitor changes in vacuolar morphology during SI. In untreated growing pollen tubes, a reticulate structure was observed throughout the pollen tube shank; this arrangement has been described in growing Arabidopsis pollen tubes by Hicks *et al.* (2004). The different vacuolar organizations observed after SI induction in the heterologous Arabidopsis ‘SI’ system were classified into different morphologies (Fig. 7A, B). We identified a series of changes to the vacuolar appearance (‘morphology B–G’, Fig 7A), with different sized globular structures formed over time. Quantification of these morphological changes showed evidence of a distinct progression over time (Fig. 7B). These data show that the genetically encoded vacuolar marker WAVE9R allows visualization of SI-induced changes to the organization of pollen tube vacuoles.

### PrpS–PrsS interaction triggers a DEVDase/caspase-3-like activity and cell death

**Fig. 8.**
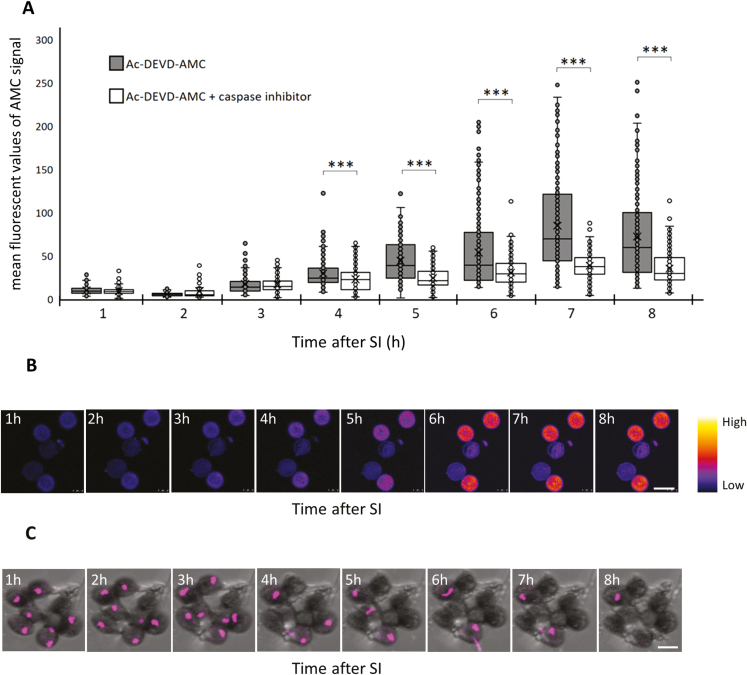
SI induction activates a DEVDase/caspase-3-like activity and nuclear disruption in Arabidopsis pollen. (A) Quantification of AMC fluorescence provides a measure of DEVDase/caspase-3-like activity in pollen grains during the SI response in the absence/presence of the caspase-3 inhibitor Ac-DEVD-CHO (*n*=100–350). (B) Representative time-lapse images showing the increase in the AMC fluorescent signal in pollen grains after SI induction. Scale bar=20 µm. (C) Nuclear disruption after SI induction indicated by the loss of NLS–tdTomato signal (magenta) in the nucleus. Scale bar=20 µm.

Proteases exhibiting caspase-like activities have been implicated in many PCD-related events in plants (Sueldo and van der Hoorn, 2017). Previous studies showed that SI-induced PCD in *Papaver* involves a DEVDase/caspase-3-like activity (Thomas and Franklin-Tong, 2004; Bosch and Franklin-Tong, 2007) and that pre-treatment of pollen expressing *Papaver* PrpS with the caspase-3 inhibitor Ac-DEVD-CHO reduced *S*-specific death (Thomas and Franklin-Tong, 2004; Bosch and Franklin-Tong, 2008; de Graaf *et al.*, 2012). Here we used the fluorescent probe Ac-DEVD-AMC to more directly determine if a cognate PrpS–PrsS interaction triggers a DEVDase/caspase-3-like activity in Arabidopsis lines expressing PrpS_1_. Caspase-3 cleaves the tetrapeptide, releasing the fluorogenic AMC, which can be imaged to assess DEVDase activity in pollen grains. After SI, an increase in fluorescence, indicating an increase in DEVDase activity, was observed (Fig. 8A, B). This increase in activity was significantly reduced when pollen was pre-treated with the caspase-3 inhibitor Ac-DEVD-CHO (Fig. 8A). The increase in caspase-3-like activity was significantly different compared with those pre-treated with the inhibitor from 4 h after SI induction (***P<0.001, Wilcoxon, Fig. 8A). We also used the genetically encoded fluorescent-tagged NLS–tdTomato to follow nuclear integrity and disruption in SI-induced pollen, as nuclear permeabilization is typical for apoptosis in animal cells (Taylor *et al.*, 2008). An increasing number of pollen grains lost their fluorescence signal of NLS–tdTomato in the nucleus after SI induction, indicating disruption of nuclei (Fig. 8C). Together, these data confirm that a cognate PrpS–PrsS interaction leads to a similar endpoint to *Papaver* SI-PCD in the engineered Arabidopsis ‘SI’ system, with cell death utilizing a DEVDase/caspase-3-like activity.

## Discussion

Pollination events are complex, crucial steps in plant reproduction leading to fertilization and seed set. This not only provides an intriguing system to study fundamental processes involved in tip growth (a topic of this Special Issue), but we also anticipate that a better mechanistic understanding may eventually provide tools that can be used in improving crop production. By studying cellular alterations that can stop tip growth, one can achieve insights into events controlling growth. SI in *Papaver rhoeas* triggers a signalling network resulting in inhibition of pollen tube tip growth and subsequent PCD of incompatible pollen. The development of an *in vitro* bioassay to study *Papaver* SI using *S*-specific inhibition of pollen tube growth has played a key part in advancing our mechanistic understanding of how this is achieved and has positioned the *Papaver* SI system as one of the best understood SI systems in the plant kingdom. Moreover, by uncovering a complex and elaborate network of events involved in inhibition, these studies have contributed to our understanding of how tip growth is regulated.

Although the *Papaver* SI system has provided an excellent model system to investigate cell–cell recognition, intracellular signalling, and PCD at a molecular level, the extremely limited genetic resources in this system represent a serious bottleneck. The development of transgenic Arabidopsis lines with functional *PrpS* expressed in its pollen (De Graaf *et al.*, 2012) and later functional transfer of both *Papaver* SI *S*-determinants to Arabidopsis reproduction *in planta* (Lin *et al.*, 2015) demonstrated that the two components, PrpS and PrsS, are sufficient to elicit an SI response in another species. This successful functional transfer suggests that the components involved in the SI events of *Papaver*, downstream of the cognate interaction of the *S*-determinants that triggers the SI response, may be conserved in pollen tubes and are likely to be ancient, as they could be successfully recruited in a distantly related species (Lin *et al.*, 2015). The availability of Arabidopsis plants expressing an SI response with all the key features of *Papaver* SI opens up exciting new opportunities to genetically dissect SI-induced signalling networks.

Here we have described the first use of fluorescent genetic tools such as YC3.6 to follow SI-induced alterations in [Ca^2+^]_cyt_, pHGFP to monitor [pH]_cyt_, LifeAct-mRuby2 to follow remodelling of the actin cytoskeleton, mCherry expressed at the tonoplast to visualize alterations in vacuolar morphology, and NLS–tdTomato to observe nuclear disruption. We have shown that this heterologous system is robust and allows us to perform detailed live-cell imaging of key events involved in *Papaver* SI. These results highlight the opportunities for using genetically encoded markers/probes to deepen our understanding of this important SI system. A particular strength of this transgenic system is that it facilitates investigating links between different cellular events (e.g. pH and actin dynamics) by multiparameter imaging and identifying new components involved in/affected by SI induction. Moreover, we can manipulate the speed of the response to suit what we wish to investigate by using lines with higher or lower PrpS expression levels. For instance, the use of ‘rapid’ (high expressing) lines enabled us to image and analyse the entire process of the SI-induced reorganization of the F-actin cytoskeleton, from highly dynamic long filament bundles to stable foci. Use of ‘slow’ (low expressing) lines, which more closely resemble the timing of SI-induced events reported for *Papaver* (Bosch and Franklin-Tong, 2008; Wilkins *et al.*, 2014; Wang *et al.*, 2019), facilitates capturing transient events that occur early during SI, for instance CME (discussed below), and the detailed dissection of changes in actin dynamics during the SI response.

In addition to providing higher temporal resolution of SI-induced events than were previously described in *Papaver*, genetically encoded probes can also identify new events involved. Here we provide the first evidence that CME is rapidly inhibited during SI. TPLATE interacts with clathrin (Van Damme *et al.*, 2011) and is part of a unique multisubunit protein complex (TPLATE complex, TPC), which is connected to the early events of CME at the plasma membrane in concert with the adaptor protein complex 2 (AP-2) (Van Damme *et al.*, 2011; Gadeyne *et al.*, 2014). The dislodging of TPLATE from the plasma membrane and the inhibition of FM4-64 internalization soon after SI induction (Fig. 2A–D) provide strong evidence that the CME machinery is disrupted early on (within the first few minutes) during the SI response. The dissipation of TPLATE and CLC2 from the plasma membrane after treatment with propionic acid at low pH (pH 5.5) is in agreement with earlier work on Arabidopsis root cells where CME was inhibited by cytoplasmic acidification (Dejonghe *et al.*, 2016). Moreover, caffeine addition to dividing tobacco BY-2 cells has been shown to dislodge TPLATE and clathrin from the growing cell plate within minutes after caffeine addition (Van Damme *et al.*, 2011). Caffeine is linked to the modulation of [Ca^2+^]_cyt_, and [Ca^2+^]_cyt_ has been shown to inhibit phospholipid [PI(4,5)P_2_] recognition by PH domain-containing proteins (Bilkova *et al.*, 2017). As negatively charged phospholipids are involved in adaptor complex recruitment in animal cells (Kelly *et al.*, 2014), the dislodgement of the endocytic machinery from the plasma membrane of the pollen tubes might therefore be caused by a combined action of acidification as well as SI-induced increases in [Ca^2+^]_cyt_, which occur prior to the inhibition of pollen tube growth (Franklin-Tong *et al.*, 1993, 1997). Together with the rise in [Ca^2+^]_cyt_ and the depolymerization/reorganization of the actin cytoskeleton, the disruption of CME represents another very early event that may contribute to, or be a consequence of, the rapid pollen tube growth inhibition after SI. The Arabidopsis ‘SI’ system provides us with the genetic tools to dissect cause and effect regarding CME inhibition and pollen tube growth inhibition, and to investigate potential links between CME, pH, and the actin cytoskeleton.

These studies have also revealed, for the first time, details about the early SI-induced dynamic reorganization of F-actin filament bundles over time. Previously, we only had images of a few fixed time points during SI. Now we have been able to begin to visualize reorganization of filaments into different arrays and fragmentation and shortening of filaments in real time, suggesting that severing may be involved at the earlier stages. Later after SI induction, kymograph analyses revealed that F-actin adjacent to the plasma membrane suddenly alters from being highly dynamic to quite static. The organization and regulation of the actin cytoskeleton have been thoroughly studied in pollen tubes and proven to be crucial for pollen tube growth (reviewed by Qu *et al*., 2015). A set of ABPs have been found to be important for the spatial distribution and dynamics of the actin cytoskeleton (reviewed by Staiger *et al.*, 2010). Amongst these ABPs, at least two are associated with SI-induced actin foci: ADF7 and CAP1 (Poulter *et al.*, 2010). ADF7 severs actin filaments and promotes normal pollen tube growth by mediating actin filament turnover (Zheng *et al.*, 2013). CAP1 is a major player in regulating actin turnover via its nucleotide exchange activity by synergizing with ADF and profilin (Jiang *et al.*, 2019). Intriguingly, it has been established that the activities of these two ABPs are pH sensitive (Carlier *et al.*, 1997; Gungabissoon *et al.*, 1998; Allwood *et al.*, 2002; Chen *et al.*, 2002; Lovy-Wheeler *et al.*, 2006). The SI-induced drop in [pH]_cyt_ may therefore alter the activities of these ABPs, which may contribute to the observed dramatic reorganization of the actin cytoskeleton through alterations of actin dynamic events such as nucleation, severing, bundling/debundling, aggregation, etc. The Arabidopsis ‘SI’ lines dual-labelling actin and other factors provide us with powerful tools to explore the mechanistic links between the actin cytoskeleton and other regulatory events in *Papaver* SI.

In the future, the availability of transgenic ‘SI’ Arabidopsis plants with all the key features of the *Papaver* SI system will allow us to overcome the bottleneck of limited genetic resources of *P. rhoeas*. Full forward and reverse genetics toolboxes available for this model plant, such as CRISPR or T-DNA mutant lines, gene silencing/overexpression lines, and lines with fluorescent markers, can now be exploited for further dissecting the molecular mechanisms involved in SI-PCD. These approaches could be employed to identify new factors involved in *Papaver* SI-PCD, especially those that are important to tip growth, as well as the components involved in the later ‘execution’ phase of the SI-induced PCD process. In summary, using genetically encoded fluorescent probes in combination with live-cell imaging, we were able to confirm and evaluate a range of early events known to be crucial for *Papaver* SI (increase in [Ca]_cyt_, decrease in [pH]_cyt_, and alterations of the actin cytoskeleton), as well as later events (alterations of vacuolar morphologies and PCD). In addition, we identified CME inhibition as a new event affected early on by SI. As such, this study has provided further evidence of the suitability and opportunities that the heterologous Arabidopsis ‘SI’ system provides to further our understanding of SI-PCD in *Papaver* and for testing new hypotheses about the cellular mechanisms and genetic components involved in the SI-PCD response and tip growth of plant cells.

## Supplementary data

Supplementary data are available at *JXB* online.

Movie S1. Growth arrest of an Arabidopsis pollen tube after SI induction.

Movie S2. F-actin reorganization and foci formation during the SI response.

Supplementary Protocols. Construction of transgenic Arabidopsis lines.

Fig. S1. Diagram of the transgene cassettes that were used for transformation of *Arabidopsis thaliana* in this study.

Fig. S2. TPLATE–TagRFP localizes at the plasma membrane.

Table S1. Overview of transgenic Arabidopsis lines used in this study.

Table S2. Oligonucleotides used for transgene construction and expression analysis.

eraa092_suppl_supplementary_figures_S1_S2_tables_S1_S2_Prtotocols

eraa092_suppl_supplementary_video_S1

eraa092_suppl_supplementary_video_S2
