# Supplementary material for: New opportunities and insights into Papaver self-incompatibility by imaging engineered Arabidopsis pollen
Source: J Exp Bot. 2020 Feb 26;71(8):2451–63. doi: 10.1093/jxb/eraa092 (PMC7178406; doi:10.1093/jxb/eraa092)
Supplement: eraa092_suppl_supplementary_figures_S1_S2_tables_S1_S2_Prtotocols [file eraa092_suppl_supplementary_figures_s1_s2_tables_s1_s2_prtotocols.pdf]

New opportunities and insights into *Papaver* self-incompatibility by imaging engineered Arabidopsis pollen

Ludi Wang, Marina Triviño, Zongcheng Lin, José Carli, Deborah J. Eaves, Daniël Van Damme, Moritz K. Nowack, Vernonica E. Franklin-Tong, Maurice Bosch

Supplementary data

Movie S1. Growth arrest of transgenic Arabidopsis pollen tube after the SI induction.

Movie S2. F-actin reorganisation and foci formation during the SI response.

Supplementary Protocols. Construction of transgenic Arabidopsis lines.

Figure S1. Diagram of the transgene cassettes that were used for transformation of *Arabidopsis thaliana* in this study.

Figure S2. TPLATE-TagRFP localizes at the plasma membrane.

Table. S1. Overview of transgenic Arabidopsis lines used in this study.

Table. S2. Oligonucleotides used for transgene construction and expression analysis.

Movie S1. Growth arrest of transgenic Arabidopsis pollen tube after the SI induction.

Time lapse imaging (1 fps) of two pollen tubes (both expressing *PrpS$_1$*) growing *in vitro*. Upper panel: ‘mock’ treated by addition of GM; lower panel: treatment by addition of recombinant PrsS$_1$ (an incompatible combination with PrpS$_1$) results in rapid inhibition of growth.

Movie S2. F-actin reorganisation and foci formation during the SI response.

Confocal time lapse imaging (15 frames / min) of a pollen tube (Line 7, Table S1) growing *in vitro* with addition of recombinant PrsS$_1$ (at time 00:00). Longitudinal F-actin arrays of filament bundles in the shank of the pollen tube are arranged longitudinally, parallel to the growth axis. After SI induction (t = 00:00), F-actin dynamics altered and the formation of punctate F-actin foci was observed. This is the first time we have observed the dynamic changes to the cytoskeleton using live-cell imaging.

Supplementary Protocols: Construction of transgenic Arabidopsis lines

Transgenic lines coexpressing *pHGFP* or *YC3.6* with *PrpS$_1$*

The dual-expression clones *pNTP303::PrpS$_1$-pNTP303::YC3.6*, and *pNTP303::PrpS$_1$-pNTP303::pHGFP* were generated using GreenGate cloning (Lampropoulos *et al*., 2013). High-fidelity Phusion DNA polymerase (New England BioLabs) was used for the amplification of all the DNA fragments. The *ntp303* promoter was amplified using primer sets *F-A-pNTP303/R-B-pNTP303* or *F-D-pNTP303/R-E-pNTP303* with the genomic DNA of a transgenic line expressing PrpS$_1$-GFP (de Graaf *et al*., 2012) as template. The resulting PCR fragments were cloned into *pJET1.2* using the CloneJET PCR Cloning Kit (ThermoFisher) to obtain the entry vectors *pEN-A-pNTP303-B* and *pEN-D-pNTP303-E*. Similarly, *pEN-B-PrpS$_1$-C* was generated by cloning of the *PrpS$_1$* DNA fragment amplified using primers *F-B-PrpS$_1$/R-C-PrpS$_1$* into *pJET1.2*. To create entry vectors for the terminator *RBCS* (*tRBCS*), yellow cameleon *YC3.6*, and *tMAS* (terminator of mannopine synthase), the corresponding DNA fragments were amplified using primer sets *F-C-tRBCS/R-D-tRBCS*, *F-E-YC3.6/R-F-YC3.6* and *F-F-tMAS/R-G-tMAS* with expression vector *pUBQ10::NLS-YC3.6* (Krebs *et al*., 2012) as template. The resulting PCR fragments were cloned into *pJET1.2* to obtain entry vectors *pEN-C-tRBCS-D, pEN-E-YC3.6-F* and *pEN-F-tMAS-G*. *pHGFP* was amplified using primers *F-E-pHGFP/R-F-pHGFP* with the genomic DNA of transgenic line *pUBQ10::pHGFP* (Fendrych *et al*., 2014) as template, and subsequently cloned into *pJET1.2* to obtain *pEN-E-pHGFP-F*. These entry clones were cloned into the GreenGate destination vector *pFAST-RK-AG* (PSB-VIB GreenGate cloning collection) to obtain the dual-expression vectors *pNTP303::PrpS$_1$-pNTP303::YC3.6* and *pNTP303::PrpS$_1$-pNTP303::pHGFP*. Detailed primer information can be found in Table S2. The expression vectors were transformed into GV3101 *Agrobacterium tumefaciens* competent cells. The floral-dipping method was adopted to stably transform Col-0 Arabidopsis plants as described previously (Fendrych *et al*., 2014).

Transgenic lines with the *pNTP303::Lifeact-mRuby2_pNTP303::PrpS$_1$-GFP* background

Promoter *NTP303* was amplified from template vector *pORE3-NTP303::PrpS$_1$* using primers *KpnI-pNTP303F* and *BglII-pNTP303R* (Table S2). The *LifeAct-mRuby2-tnos-3* fragment was amplified from template vector *pTZUBI::Lifeact-mRuby2* using primers *SalI-LifeAct F* and *HindIII-tnos R2* (Table S2) using GoTaq®Green Master Mix (Promega, UK). PCR products were captured using pGEM®-T Vector System (Promega). Inserts were excised and

sequentially transferred into *pLH6000* (DNA Cloning Service e.K. Germany). The resultant expression vector *pLH6000-pNTP303::Lifeact-mRuby2-tnos-3* was transformed into *Agrobacterium tumefaciens* GV3101 and used to stably transform *Arabidopsis thaliana* expressing *PrpS*$_1$*-GFP* (de Graaf *et al*., 2012) with *Lifeact-mRuby2* by floral dipping method (Fendrych *et al*., 2014).

Transgenic lines *pNTP303::ADF7-mTagBFP2* and *pNTP303::CAP1-mTagBFP2* coexpressing *Lifeact-mRuby* and *PrpS*$_1$*-GFP*

The pollen-specific promoter *npt303* and the blue fluorescent tag *mTagBFP2* (Pasin *et al*., 2014) were amplified by PCR with Q5 polymerase (New England Biolabs) using the template vectors *pNTP303::Lifeact-mRuby2_pNTP303::PrpS*$_1$*-GFP* and *pEN-L1-mTagBFP2-L2* (unpublished material obtained from Steffen Vanneste) respectively, and introduced into *pDONRP4-P1R* (promoter) and *pDONR P2R-P3* (tag) entry vectors by means of Gateway cloning. Similarly, CAP1 and ADF7 coding sequences were amplified by PCR using *A. thaliana* Col-0 cDNA as template and introduced into the pDONR 207 entry vector. Detailed primer information can be found in Table S2. Final constructs were obtained by three-fragment recombination (Life Technologies), into the *pB8m34GW* Fast Green destination vector (Karimi *et al*., 2007). Plants expressing *CAP-mTagBFP2* and *ADF7-mTagBFP2* under the pollen specific promoter *npt303* were generated by transforming Arabidopsis plants with the *pNTP303::Lifeact-mRuby2_ pNTP303::PrpS*$_1$*-GFP* background.

Transgenic lines expressing *PrpS*

Arabidopsis lines expressing *PrpS* (without fluorescent tag) under control of the *NTP303* promoter (*pNTP303::PrpS*) were generated by transformation of *A. thaliana* Col-0 with tumor inducing (Ti) vectors *pORE3-NTP303::PrpS*$_{1/3}$ based on the protocols published by Davis *et al*. (2009) and Zhang *et al*. (2006). Target DNA fragments for *pNTP303::PrpS*$_{1/3}$ were amplified using the template vectors *pGreen0029-pNTP303::PrpS*$_{1/3}$*-GFP* (de Graaf *et al*., 2012) and primers with SacII and EcoRI restriction sites flanked in the 5’ and 3’ ends respectively (Table S2).

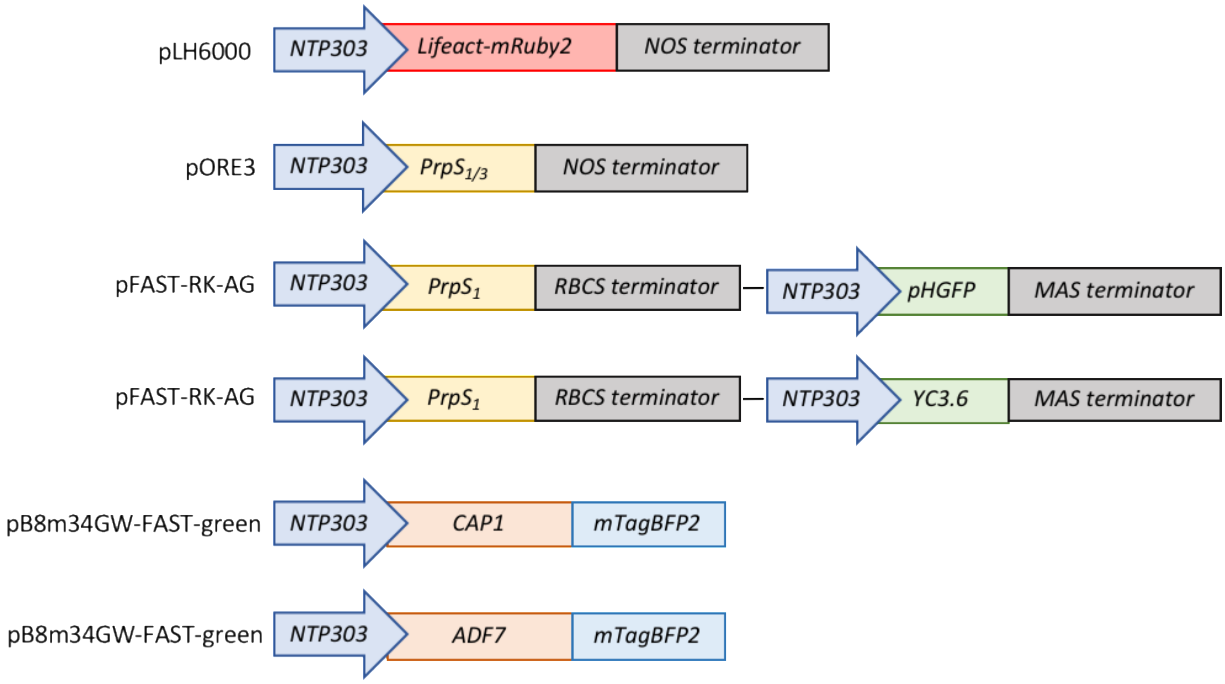

Figure S1. Diagram of the transgene cassettes that were used for transformation of *Arabidopsis thaliana* in this study.

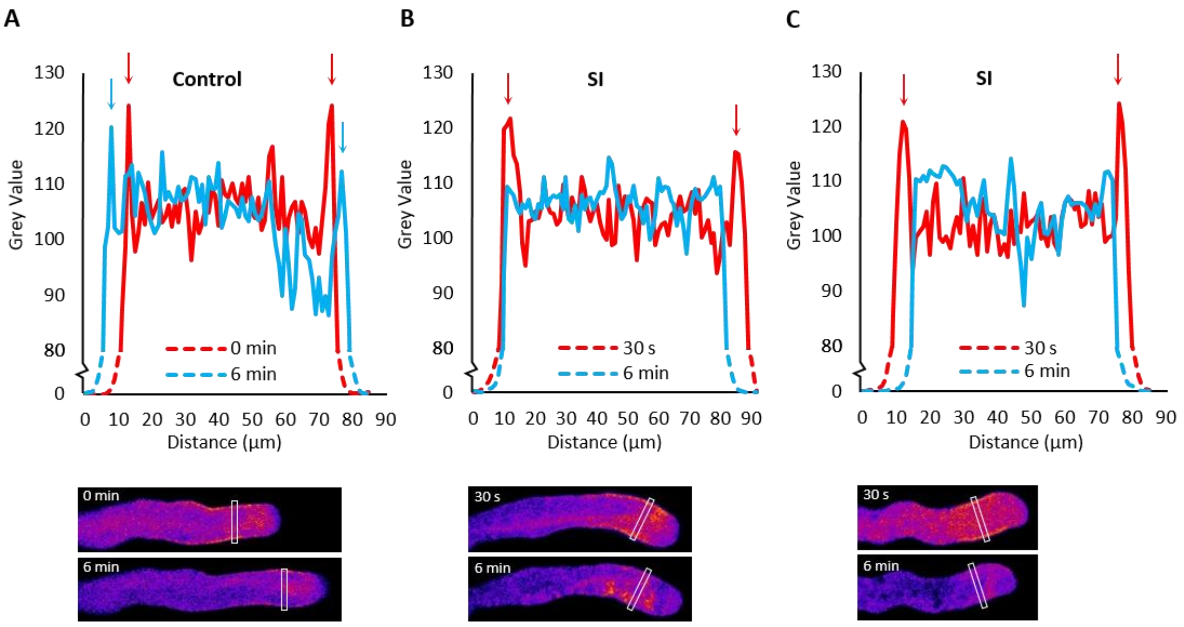

Figure S2. TPLATE-TagRFP localizes at the plasma membrane. Fluorescence intensity profiles of the TPLATE-TagRFP signal (see also Figure 2) were taken across the white boxes indicated in the representative pollen tube images. Arrows with the corresponding colour indicate the localization of TPLATE on the plasma membrane after treatment with growth medium (control, A) or SI induction (B and C). For control tubes, plasma membrane localization is evident at both 0 min and 6 min. While plasma membrane localization of TPLATE-TagRFP can be observed 30 s after SI induction, this is not the case after 6 min.

Table S1. Overview of transgenic Arabidopsis lines used in this study.

(A) Transgene cassettes present in each line. Constructs containing *PrpS₁* are in bold.

(B) Classification of *PrpS₁* expression levels and SI response for the *PrpS₁* expressing Arabidopsis lines used in this study.

A

Lines	Constructs
1	***pNTP303::PrpS₁-GFP*** (de Graaf *et al.*, 2012)
2	*pNTP303::PrpS₃-GFP* (de Graaf *et al.*, 2012)
3[a]	*pLAT52::TPLATE (At3g01780)-TagRFP* in *tplate* mutant *SALK_003086* (unpublished; material obtained from Daniel Van Damme; Van Damme *et al.*, 2006), ***pNTP303::PrpS₁-GFP***
4	*pLAT52::TPLATE (At3g01780)-GFP, pRPS5A::CLC2 (At2g40060)-TagRFP* (Gadeyne *et al.*, 2014)
5[a]	***pNTP303::YC3.6-pNTP303::PrpS₁***, *pNTP303::Lifeact-mRuby2*
6[a]	*pNTP303::Lifeact-mRuby2*, ***pNTP303::PrpS₁-GFP***
7[a]	***pNTP303::pHGFP-pNTP303::PrpS₁***, *pNTP303::Lifeact-mRuby2*
8[b]	*pNTP303::ADF7-mTagBFP2, pNTP303::Lifeact-mRuby2*, ***pNTP303::PrpS₁-GFP***
9[b]	*pNTP303::CAP1-mTagBFP2, pNTP303::Lifeact-mRuby2*, ***pNTP303::PrpS₁-GFP***
10[a]	*pUBQ10::VAMP711 (At4g32150)-mCherry* (Geldner *et al.*, 2009), ***pNTP303::PrpS₁-GFP***
11	***pNTP303::PrpS₁***
12[a]	*pUBQ10::NLS-tdTomato* (Shaner *et al.* 2004), ***pNTP303::PrpS₁-GFP***

a, Combination of cassettes was generated by crossing; b, Combination of cassettes was generated by transformation

B

PrpS₁ constructs	PrpS₁ expression level/SI response	Corresponding figures
PrpS₁-GFP (line 1-3, 6, 8-10, 12)	Low/Slow	Figure 1B, 2A-D, 4A, 4C-D, 4F-G, 6, 7, 8C
YC3.6_PrpS₁ (line 5)	High/Rapid	Figure 3, 4B, 4E
pHGFP_PrpS₁ (line 7)	High/Rapid	Figure 5
PrpS₁ (line 11)	Low/Slow	Figure 8A-B

Table S2. Oligonucleotides used for transgene construction (A) and expression analysis (B). *F*- forward, *R*- reverse primers.

A

Primer name	Sequence (5'-3')
F-A-pNTP303	*TTTTGGTCTCAACCTGATACACTCGCAACGTGTGTATCCTAA*
R-B-pNTP303	*TTTTGGTCTCATGTTGACGTTGTTTTTTTTATTCTTTAATCCCC*
F-B-PrpS$_1$	*TTTTGGTCTCAAACAATGGCCCGAAGTGGAAGTGTTGTTAC*
R-C-PrpS$_1$	*TTTTGGTCTCAAGCCTTAAGCTTGAGTTATAAGATGAGGGGAATC*
F-C-tRBCS	*TTTTGGTCTCAGGCTGAGCTTTCGTTCGTATCATCGGTTTC*
R-D-tRBCS	*TTTTGGTCTCACTGATCGATTGATGCATGTTGTCAATCAATTG*
F-D-pNTP303	*TTTTGGTCTCATCAGGATACACTCGCAACGTGTGTATCCTAA*
R-E-pNTP303	*TTTTGGTCTCAGCAGGACGTTGTTTTTTTTATTCTTTAATCCCC*
F-E-YC3.6	*TTTTGGTCTCACTGCGGAGTCGACTCGAGTGCGGCCGCCACC*
R-F-YC3.6	*TTTTGGTCTCATAGTCGAAAGCTCTGCAGCCAACTAGTTTAC*
F-E-pHGFP	*TTTTGGTCTCACTGCATGAGTAAAGGAGAAGAACTTTTCACTGG*
R-F-pHGFP	*TTTTGGTCTCATAGTTTATTTGTATAGTTCATCCATGCCATGTG*
F-F-tMAS	*TTTTGGTCTCAACTAAATTCCAGCTTCCCTGAAACCTTGGAC*
R-G-tMAS	*TTTTGGTCTCAATACGCCGACCAACCGCAAGCGTTGTCAGTG*
KpnI-pNTP303 F	*AAAGGTACCGATACACTCGCAACGTGTGT*
BglII-pNTP303 R	*GCGAGATCTGACGTTGTTTTTTTTATTC*
SalI LifeAct F	*AGAGTCGACATGGGTGTCGCAGATTTGAT*
HindIII tnos R2	*GCGAAGCTTCCCGATCTAGTAACATAGATGAC*
F-NTP303	*GGGGACAACTTTGTATAGAAAAGTTGGATACACTCGCAACGTGTGT*
R-NTP303	*GGGGACTGCTTTTTTGTACAAACTTGCATGACGTTGTTTTTTTTAT*
F-mTagBFP2	*GGGGACAGCTTTCTTGTACAAAGTGGCCATGTCATCTAAGGGTGAA*
R-mTagBFP2	*GGGGACAACTTTGTATAATAAAGTTGATTACGCTAAGTCTTCCTC*
F-CAP1	*GGGGACAAGTTTGTACAAAAAAGCAGGCTATGGAAGAGGATTTGATTAAG*
R-CAP1	*GGGGACCACTTTGTACAAGAAAGCTGGGTCGGCACCTGAATGCGAGACCGG*
F-ADF7	*GGGGACAAGTTTGTACAAAAAAGCAGGCTATGGCGAACGCGGCGTCGGGG*
R-ADF7	*GGGGACCACTTTGTACAAGAAAGCTGGGTCGAGAGCTCGGCTTTTGATAATG*
F-SacII-NTP303	*AAAAAAACCGCGGGATACACTCGCAACG*
R-EcoRI-PrpS$_1$	*CGGAATTCTTAAGCTTGAGTTATAAGATGAGG*

B

Primer name	Sequence (5'-3')
PrpS$_1$-F	*CCATGCCCCGAAGTGGAAGTGTTG*
PrpS$_1$-R	*CCTTAAGCTTGAGTTATAAGATGAGGGGAATCC*
GAPD-F	*CACTGACAAAGACAAGGCTGCAGC*
GAPD-R	*CCTGTTGTCGCCAACGAAGTCAG*

References

Davis, A.M., A. Hall, A.J. Millar, C. Darrah, and S.J. Davis. 2009. Protocol: Streamlined sub-protocolsfor floral-dip transformation and selection of transformants in *Arabidopsis thaliana*. Plant Methods **5**, 3.

de Graaf BH, Vatovec S, Juarez-Diaz JA, Chai L, Kooblall K, Wilkins KA, Zou H, Forbes T, Franklin FC, Franklin-Tong VE. 2012. The *Papaver* self-incompatibility pollen *S*-determinant, PrpS, functions in *Arabidopsis thaliana*. Current biology **22**, 154-159.

Fendrych, M., T. Van Hautegem, M. Van Durme, *et al*. 2014. Programmed Cell Death Controlled by ANACO33/SOMBRERO Determines Root Cap Organ Size in Arabidopsis. Current Biology **24**, 931-940.

Gadeyne A, Sánchez-Rodríguez C, Vanneste S, Di Rubbo S, *et al*. 2014. The TPLATE adaptor complex drives clathrin-mediated endocytosis in plants. Cell **156**, 691-704.

Geldner, N., V. Dénervaud-Tendon, D.L. Hyman, U. Mayer, Y.-D. Stierhof, and J. Chory. 2009. Rapid, combinatorial analysis of membrane compartments in intact plants with a multicolor marker set. The Plant Journal **59**, 169-178.

Karimi, M., A. Depicker, P. Hilson. 2007. Recombinational Cloning with Plant Gateway Vectors. Plant Physiology **145**, 1144-1154.

Krebs, M., K. Held, A. Binder, K. Hashimoto, G. Den Herder, M. Parniske, J. Kudla, K. Schumacher. 2012. FRET-based genetically encoded sensors allow high-resolution live cell imaging of Ca^{2+} dynamics. The Plant Journal **69**, 181-192.

Lampropoulos, A., Z. Sutikovic, C. Wenzl, I. Maegele, J.U. Lohmann, J. Forner. 2013. GreenGate - A Novel, Versatile, and Efficient Cloning System for Plant Transgenesis. PLOS ONE **8**, e83043.

Pasin, F., S. Kulasekaran, P. Natale, C. Simón-Mateo, J.A. García. 2014. Rapid fluorescent reporter quantification by leaf disc analysis and its application in plant-virus studies. Plant methods **10**, 22-22.

Shaner, N.C., R.E. Campbell, P.A. Steinbach, B.N.G. Giepmans, A.E. Palmer, R.Y. Tsien. 2004. Improved monomeric red, orange and yellow fluorescent proteins derived from *Discosoma* sp. red fluorescent protein. Nature Biotechnology **22**, 1567-1572.

Van Damme D, Coutuer S, De Rycke R, Bouget F-Y, Inzé D, Geelen D. 2006. Somatic Cytokinesis and Pollen Maturation in *Arabidopsis* Depend on TPLATE, Which Has Domains Similar to Coat Proteins. The Plant Cell **18**, 3502-3518.

Zhang. X, R. Henriques, S. Lin, Niu. QW, Chua. NH. 2006. Agrobacterium-mediated transformation of *Arabidopsis thaliana* using the floral dip method. Nature Protocols **1**, 641-646.
